# Multi-Omics Integrative Analysis Uncovers Molecular Subtypes and mRNAs as Therapeutic Targets for Liver Cancer

**DOI:** 10.3389/fmed.2021.654635

**Published:** 2021-05-24

**Authors:** Yi Shen, Wei Xiong, Qi Gu, Qin Zhang, Jia Yue, Changsong Liu, Duan Wang

**Affiliations:** Department of Cardiovascular Medicine, The Third Affiliated Hospital of Chongqing Medical University, Chongqing, China

**Keywords:** liver cancer, multi-omics, molecular subtype, ANXA2, CHAF1B, mRNA, therapeutic target

## Abstract

**Objective:** This study aimed to systematically analyze molecular subtypes and therapeutic targets of liver cancer using integrated multi-omics analysis.

**Methods:** DNA copy number variations (CNVs), simple nucleotide variations (SNVs), methylation, transcriptome as well as corresponding clinical information for liver carcinoma were retrieved from The Cancer Genome Atlas (TCGA). Multi-omics analysis was performed to identify molecular subtypes of liver cancer via integrating CNV, methylation as well as transcriptome data. Immune scores of two molecular subtypes were estimated using tumor immune estimation resource (TIMER) tool. Key mRNAs were screened and prognosis analysis was performed, which were validated using RT-qPCR. Furthermore, mutation spectra were analyzed in the different subtypes.

**Results:** Two molecular subtypes (iC1 and iC2) were conducted for liver cancer. Compared with the iC2 subtype, the iC1 subtype had a worse prognosis and a higher immune score. Two key mRNAs (ANXA2 and CHAF1B) were significantly related to liver cancer patients' prognosis, which were both up-regulated in liver cancer tissues in comparison to normal tissues. Seventeen genes with *p* < 0.01 differed significantly for SNV loci between iC1 and iC2 subtypes.

**Conclusion:** Our integrated multi-omics analyses provided new insights into the molecular subtypes of liver cancer, helping to identify novel mRNAs as therapeutic targets and uncover the mechanisms of liver cancer.

## Introduction

Liver cancer is the fifth largest malignant tumor and the second leading cause of cancer-related deaths worldwide (1, 2). It was estimated that 42,220 new cases and 30,200 death cases occurred in the United States in 2018 (3). The mortality of liver cancer accounts for about 6% of death cases of cancers in men and 3% of death cases in women (3). Most patients have advanced liver cancer when first diagnosed. As we all know, several potential risk factors contribute to the occurrence and development of liver cancer, including chronic hepatitis B/C virus infection, alcoholism and aflatoxin exposure (4). Under the exposure of these risk factors, genetics and epigenetic changes may be gradually accumulated, thereby leading to activation of oncogenes and inactivation of tumor suppressor genes, which in turn will lead to the occurrence of liver cancer (5, 6). Furthermore, cancers have association with an increased risk of coronary heart disease in time of the first 6 months following diagnoses (7). Despite the considerable progress over the past few decades, the prognosis of patients with liver cancer is still poor (5-year survival rate <20%) due to the high recurrence rate (8). Although extensive research has been conducted on the mechanisms of liver cancer occurrence and development, its etiology and carcinogenesis remain unclear. Considering the high morbidity and mortality of liver cancer, identification of effective markers and exploration of their potential roles have important clinical significance for early diagnosis, prevention, and control of liver cancer.

Growing multi-omics studies have confirmed that genomic and epigenomic imbalances can affect the occurrence and development of liver cancer. TCGA project provides genomic, epigenomic, transcriptomics, and proteomics of 32 human cancers. A number of data portals such as UCSC Cancer Genomics Browser (https://genome-cancer.ucsc.edu/) have been developed (9). As a key regulator of genomic and epigenomic abnormalities, CNV is significantly correlated with individual genetic variations and human genetic diversities, which may change gene expression via modulating mRNA expression and affecting transcription. Several CNVs have been found to be closely related to liver cancer. For example, Jagged1 copy number amplification indicates poor prognosis in patients with liver cancer (10). In-depth research on CNV may help understand the mechanisms and probe susceptible targets for liver cancer. Studies have shown that epigenetic changes such as DNA methylation, contribute to the development of liver cancer (11, 12). DNA methylation has been considered as a useful biomarker for early diagnosis of liver cancer. During carcinogenesis, abnormal DNA methylation is mainly manifested by focal methylation around the promoters of specific genes, and global methylation in non-promoter regions (13, 14). Hypermethylation of the promoter region is a crucial process that can lead to epigenetic silencing of tumor suppressor genes (15, 16). Moreover, abnormal DNA methylation of non-promoter elements is in association with intratumor heterogeneity (17).

Herein CNV, DNA methylation, as well as mRNA levels were detected in a variety of liver cancer samples. Copy number variation-correlated (CNVcor) as well as methylation-correlated (METcor) genes were identified to distinguish molecular subtypes of liver cancer. Furthermore, specific biomarkers were proposed to drive the classification of these subtypes.

## Materials and Methods

### Data Collection

HTSeq—counts and HTSeq—FPKM gene expression RNA-seq, Illumina Human Methylation 450K, and SNV data (mutect2) were downloaded from the TCGA-liver hepatocellular carcinoma (LIHC) dataset (*n* = 363) using the Genetic Disease Control (GDC) Data Portal (https://portal.gdc.cancer.gov/). The hub is last updated on 2019-08-28. Masked Copy Number Segment data were also obtained from the GDC dataset. Furthermore, clinical information of all samples including age, gender, survival status, pathologic TNM, tumor stage and overall survival time was retrieved from the TCGA data portal, listed in Table 1.

**Table 1:** Clinical characteristic information for the LIHC cohort (overall=363).

**Characteristics**	**Groups**	**Number**
Age (median [IQR])		61.00 [52.00, 69.00]
Gender (%)	Female	118 (32.5)
	Male	245 (67.5)
Race (%)	American Indian or Alaska native	1 (0.3)
	Asian	154 (42.4)
	Black or African American	17 (4.7)
	White	181 (49.9)
HBV/HCV infection status (%)	Yes	151 (41.6)
	No	212 (58.4)
Status (%)	Dead	233 (64.2)
	Alive	130 (35.8)
Pathologic T (%)	T1	179 (49.6)
	T2	91 (25.2)
	T3	77 (21.3)
	T4	13 (3.6)
	TX	1 (0.3)
Pathologic N (%)	N0	248 (68.5)
	N1	3 (0.8)
	NX	111 (30.7)
Pathologic M (%)	M0	262 (72.2)
	M1	3 (0.8)
	MX	98 (27.0)
Tumor stage (%)	I	169 (49.9)
	II	84 (24.8)
	III	82 (24.2)
	IV	4 (1.2)

### Data Preprocessing

By applying GISTIC2.0, this study calculated the genetic copy number changes for each sample (18). The methylation data preprocessing was as follows. Methylation sites that were undetectable in over 70% of specimens were removed. The KNN was then utilized for filling in missing values. Furthermore, we removed the following methylation data: (1) the methylation data of the SNP sites, (2) the methylation site data on the sex chromosome, and (3) the multi-aligned methylation site data. Methylated sites in the 200 bp range upstream or downstream from gene transcription start were retained in this study. For mRNA expression profiles, this study filtered out mRNAs with FPKM value < 0.1 across 50% specimens.

### Correlation Analysis

The correlation coefficient of CNV data or methylation data with gene expression was calculated, which was then transformed to z-value based on ln [(1 + r)/(1 – r)]. Under the screening criterion of *p* < 0.01, CNVcor and METcor genes were obtained with the test of correlation coefficient.

### Integrative Analysis of CNV, Methylation and mRNA Expression Data

Multi-omics clustering analysis was conducted by integrating CNV, methylation as well as mRNA expression profiles using the non-negative matrix factorization (NMF) package in R (19). Lambda values were used to determine optimal weights for CNV, methylation, and mRNA expression data sets.

### Immune Infiltration Estimation

Immune infiltrates across liver cancer were from the Tumor Immune Estimation Resource (TIMER) website (https://cistrome.shinyapps.io/timer/) (20, 21). The infiltration levels of six immune cells composed of B cells, CD4+ T cells, CD8+ T cells, neutrophils, macrophages, and dendritic cells were estimated.

### Gene Set Variation Analysis (GSVA)

The GSVA algorithm was applied for evaluating the enriched signaling pathways between subtypes based on gene expression profiles (22). The pathway enrichment score of each sample was determined and the differences between subtypes were analyzed by employing the limma package in R (23).

### RT-qPCR

Total RNA was extracted from 20 pairs of liver cancer tissues and normal tissues using Trizol reagent (Invitrogen, USA), which was reverse transcribed cDNA. All patients provided written informed consent. This study was approved by the Ethics Committee of The Third Affiliated Hospital of Chongqing Medical University (2019063). SYBR fluorescence quantitative PCR kit (Takara, Japan) was utilized to perform PCR. ANXA2: 5′-GTGGTGGAGATGACTGAAGCC-3′ (forward) and 5′-CCACGGGGACTGTTATTCG-3′ (reverse); CHAF1B: 5′-CCTGGAAAAGCCACTCTTGCTG-3′ (forward) and 5′- ACAGAAGCACGGAATCCTCCGA-3′(reverse); GAPDH: 5′-TGACTTCAACAGCGACACCCA-3′ (forward) and 5′-CACCCTGTTGCTGTAGCCAAA-3′ (reverse). GAPDH served as a reference control. Relative ANXA2 and CHAF1B expression was determined with the 2^−Δ*ΔCt*^.

### Western Blot

RIPA lysis buffer (Beijing Biotech Biotechnology Company, China) was used to extract total protein from tissue specimens. The protein concentration was determined with BCA assay kit (BioTek, USA). Twenty micro gram total protein was separated by 10% sodium dodecyl sulfate-polyacrylamide (SDS-PAGE) (Beyotime, Shanghai, China), and transferred to PVDF membrane (Millipore, USA). The PVDF membrane was blocked with 10% skimmed milk powder for 1 h at room temperature and incubated with the primary antibodies against ANXA2 (1/10000; ab178677; Abcam, USA), CHAF1B (1/10000; ab109442; Abcam, USA) and β-actin (1/5000; ab179467; Abcam, USA) overnight at 4°C. The membrane was washed 3 times with TBST and incubated with secondary antibody (1/3000, ab6789; Abcam, USA) for 2 h at room temperature. The membrane was visualized with an enhanced chemiluminescence solution (Thermo Fisher, USA).

### Statistical Analysis

All analyses were carried out using R packages and Graphpad Prism software. ANXA2 and CHAF1B expression was validated in liver cancer and normal tissues using the gene expression data from the International Cancer Genome Consortium (ICGC; http://icgc.org/). Each experiment was repeated three times. Data were presented as the mean ± standard deviation. Student's t test was applied for comparisons between two groups. *P* < 0.05 indicated statistical significance.

## Results

### Screening CNVCor/METCor Genes in Liver Cancer

**Figure 1:**
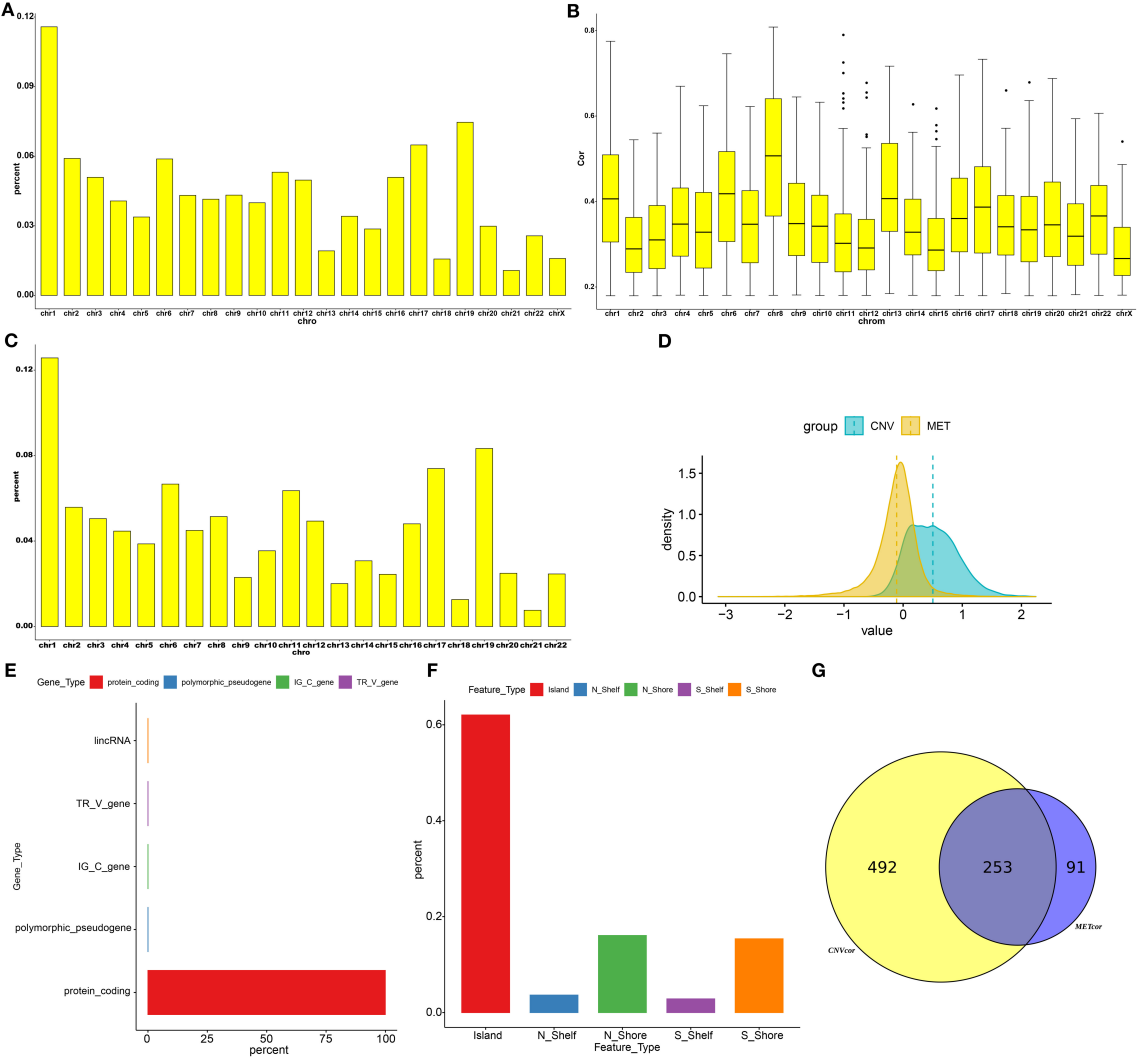
Screening CNVCor and METCor genes in liver cancer. Chromosomal distributions **(A)** and correlations **(B)** of CNVcor genes. Horizontal axis indicates chromosomes; ordinate axis represents the proportion or the correlation coefficients of CNVcor genes. The middle line of the box plot is the median of the data, which represents the average level of the sample data. The upper and lower limits of the box plot are the upper quartile and the lower quartile of the data. Black dots represent outliers. **(C)** Chromosomal distribution of METcor genes. Y-axis represents the proportion of METcor genes. **(D)** Distribution of z-values for CNVcor genes and METcor genes. Horizontal axis is z value correlation, and ordinate axis is the density distributions corresponding to z values. **(E)** The proportion of each METcor gene type. **(F)** The proportion of each methylation locus. **(G)** Venn diagram showing overlapping survival-related CNVcor genes and METcor genes.

Totally, 9161 CNVCor genes were identified (*p* < 0.01; Supplementary Table 1). As depicted in the distribution of CNVCor genes on chromosomes, CNVCor genes most frequently occurred on chr1 (FDR<0.05; Figure 1A and Table 2). The box plots showed the distribution in pearson correlation coefficients of CNVCor genes on chromosomes (Figure 1B). 16037 methylation sites corresponding 6181 genes were identified under the screening criteria of *p* < 0.01 (Supplementary Table 2). As shown in Figure 1C and Table 2, METcor genes were prone to appear on chr1. In the correlation z-value distribution, the correlation between CNVcor gene and its expression leaned to the right, while the correlation between METcor gene and its expression leaned to the left, indicating positive associations between CNVs and gene expressions, while negative associations between methylations and gene expressions (Figure 1D). METcor genes mainly contained protein-coding genes (Figure 1E). Furthermore, methylation loci were mainly situated in the island, S shore, N shore, S shelf as well as N shelf regions (Figure 1F). According to the median expression value of CNVCor/METCor genes, the samples were divided into high- and low- groups. Kaplan-Meier survival analysis was then performed. CNVCor genes and METCor genes with *p* < 0.01 were identified as survival-related CNVCor (*n* = 745)/METCor genes (*n* = 344). Two-hundred and fifty-three overlapping CNVcor genes and METcor genes were in significant association with survival of liver cancer (Figure 1G), which were used for further analysis.

**Table 2:** Fisher significance test of CNVCor and METCor gene frequencies on chromosomes.

**Chromosomes**	**All genes**	**METcor genes**	**CNVcor genes**	**METcor FisherP**	**METcor FDR**	**CNVcor FisherP**	**CNVcor FDR**
chr1	2059	777	1055	1.92E-04	0.001056215	0.011311298	0.045345148
chr10	734	219	364	0.418195975	0.460015573	0.290234004	0.35473045
chr11	1318	393	484	0.267306507	0.322891157	1.95E-05	4.28E-04
chr12	1030	305	453	0.278860544	0.322891157	0.390088389	0.451681293
chr13	323	124	175	0.084400506	0.142831625	0.098603748	0.216928245
chr14	820	190	311	6.67E-05	4.89E-04	0.003285193	0.018068562
chr15	613	151	261	0.004556993	0.011139317	0.284006963	0.35473045
chr16	865	297	464	0.278179575	0.322891157	0.012366858	0.045345148
chr17	1187	457	591	0.001112523	0.00407925	0.156199369	0.286365511
chr18	271	78	143	0.450440412	0.471889955	0.220911045	0.35473045
chr19	1461	515	680	0.060434835	0.110797198	0.904106072	0.904106072
chr2	1296	345	538	0.003469529	0.010615833	0.040472584	0.111299606
chr20	540	154	272	0.242389855	0.322891157	0.267280512	0.35473045
chr21	233	47	98	0.003860303	0.010615833	0.475549326	0.523104258
chr22	489	152	234	0.815424881	0.815424881	0.685593908	0.718241237
chr3	1072	312	464	0.175300624	0.257107583	0.247938803	0.35473045
chr4	747	276	371	0.044594131	0.089188261	0.279538529	0.35473045
chr5	870	239	308	0.044592458	0.089188261	6.12E-05	6.73E-04
chr6	1043	412	536	4.37E-04	0.001922587	0.055334401	0.13526187
chr7	969	278	393	0.135649877	0.213164093	0.031565197	0.099204905
chr8	670	318	378	2.69E-08	2.96E-07	0.002640408	0.018068562
chr9	774	142	394	3.04E-10	6.68E-09	0.13166299	0.263325981

### Correlations Between CNVs and Methylations in Liver Cancer

**Figure 2:**
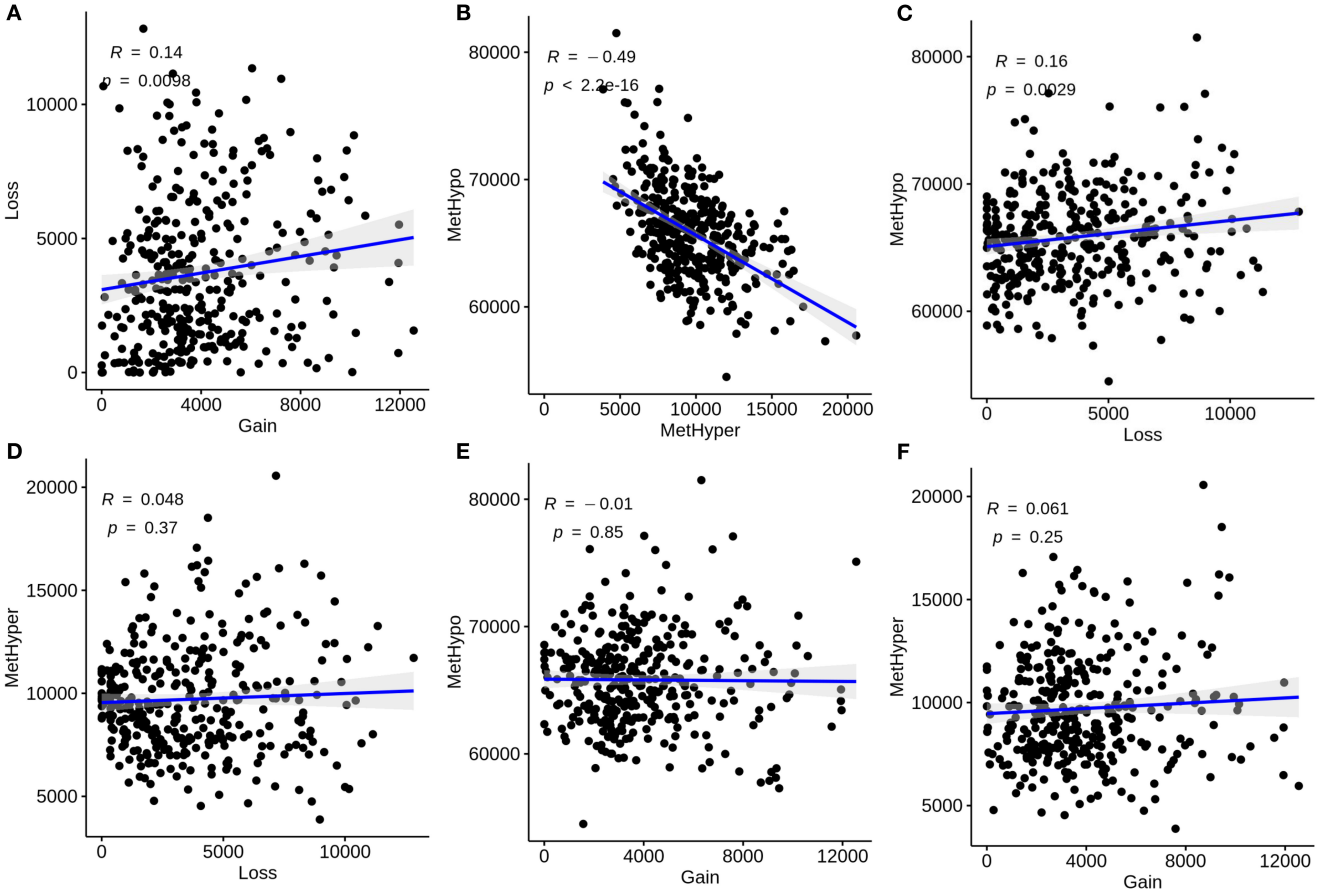
Correlation between CNVs and methylations in liver carcinoma. **(A)** Correlations of CNV gain with loss. **(B)** Correlations of MetHypo with MetHyper. **(C)** Correlations of CNV loss with MetHypo. **(D)** Correlations between CNV loss and MetHyper. **(E)** Correlations of CNV gain with MetHyper. **(F)** Correlations of CNV gain with MetHyper. X axis represents CNV or methylation scores and y axis represents CNV or methylation scores.

We further analyzed the correlations between CNVs and methylations in liver cancer. CNVs were divided into three classifications: loss, normal, as well as gain according to−0.3-0.3. We classified methylations into hypomethylation (MetHypo), normal and hypermethylation (MetHyper) based on the cutoffs of 0.2 and 0.8. The correlations among loss, gain, MetHypo and MetHyper were analyzed. The results showed that CNV gain was positively correlated to CNV loss (R = 0.14, *p* = 0.0098; Figure 2A). Furthermore, a strong negative correlation between MetHypo and MetHyper was found in Figure 2B (R = −0.49; *p* < 2.2e-16). Intriguingly, we found that CNV loss was positively correlated with MetHypo (R = 0.16, *p* = 0.0029; Figure 2C). However, there were no distinct correlations between CNV loss and MetHyper (Figure 2D), between CNV gain and MetHyper (Figure 2E), between CNV gain and MetHyper (Figure 2F).

### Identification of CNVcor and METcor Gene Molecular Subtypes

**Figure 3:**
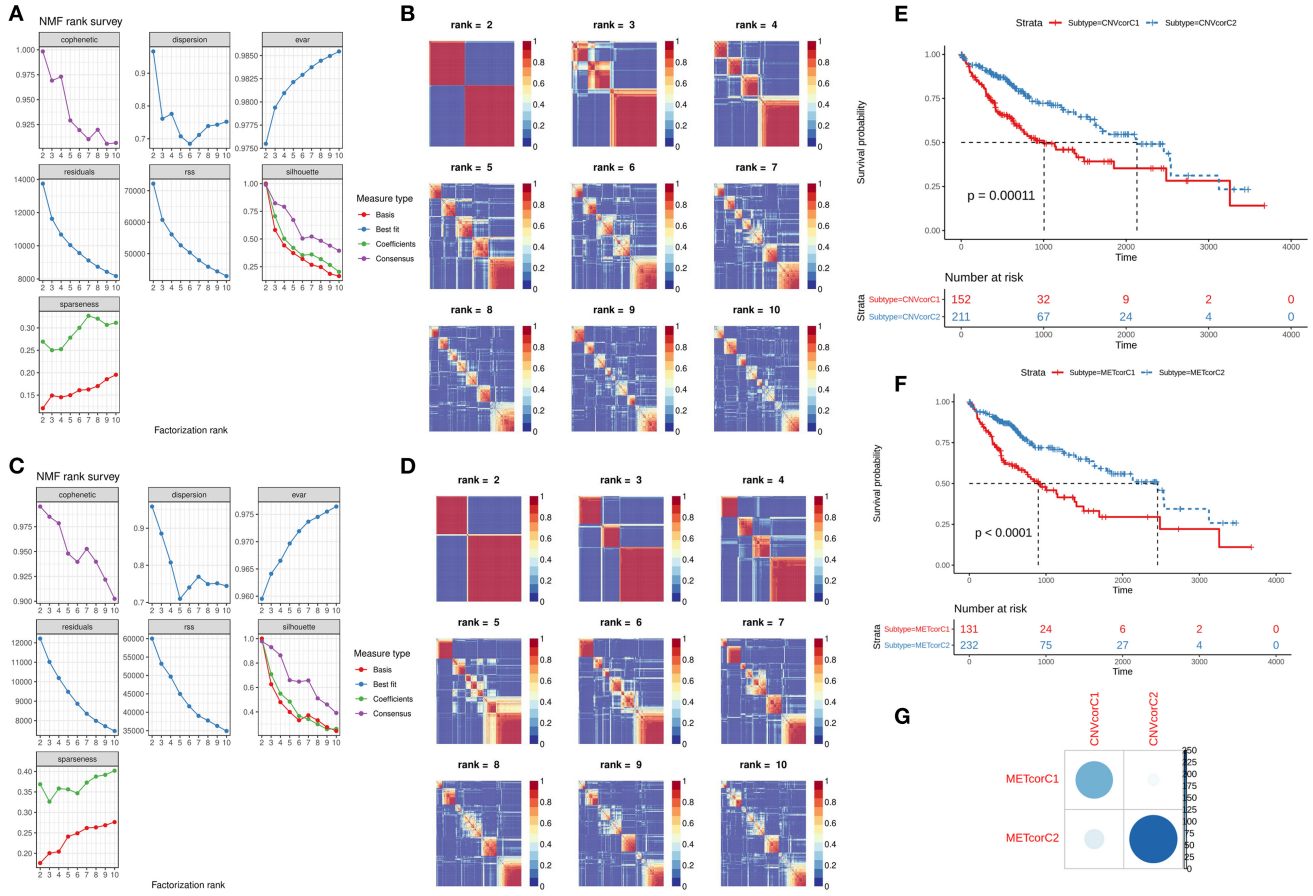
Identification of molecular subtypes according to CNVcor and METcor genes. **(A,B)** NMF cluster analysis based on CNVcor genes. **(C,D)** NMF cluster analysis based on METcor genes. Kaplan-Meier curve analysis of CNVcor gene clusters **(E)** and METcor gene clusters **(F)**. **(G)** Overlap between CNVcor and METcor gene clusters. The color shade indicates the number of overlapping specimens.

NMF method was used for clustering analysis according to CNVcor and METcor genes. The optimal number of clustering was 2 for CNVcor genes (Figures 3A,B) and METcor genes (Figures 3C,D). Both the CNVcor genes (*p* = 0.00011) and METcor genes (*p* < 0.0001) in the two molecular subtypes were in significant association with overall survival of patients with liver cancer (Figures 3E,F). We further compared the differences between CNVcor and METcor gene molecular subtypes. There were high proportions of overlapping samples between CNVcor and METcor gene molecular subtypes (Figure 3G).

### Construction of Two Multi-Omics Molecular Subtypes for Liver Cancer After Integration of CNV, DNA Methylation and mRNA Expression

**Figure 4:**
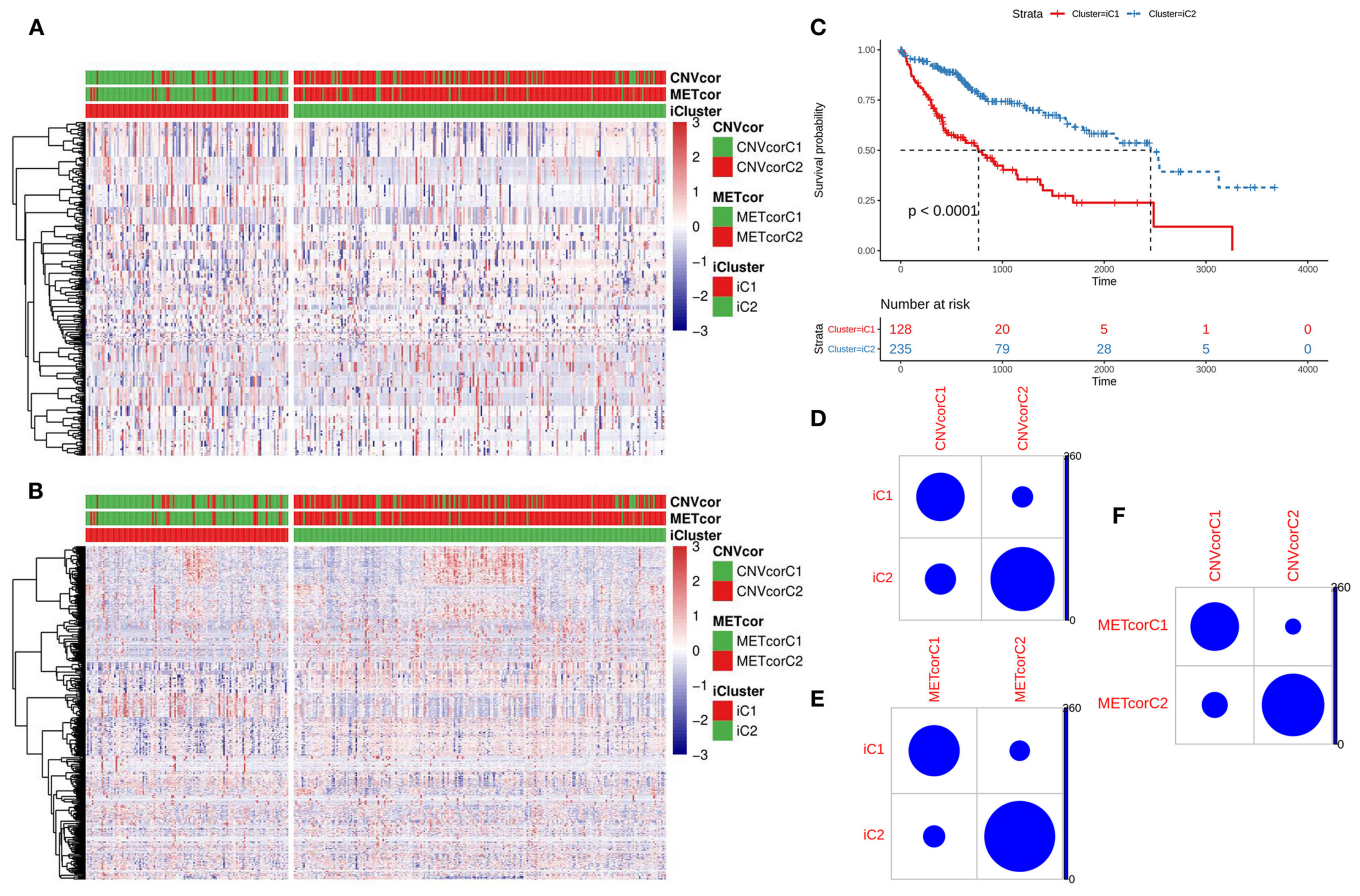
Multi-omics clustering analysis of CNV, DNA methylation and mRNA expression. **(A)** iCluster clustering heat map showing the CNV distribution of CNVcor genes. **(B)** iCluster clustering heat map showing the methylation site distribution of METcor genes. **(C)** Kaplan-Meier survival analysis results for two subtypes. **(D)** Intersection of NMF and iCluster CNVcor gene sets. **(E)** Overlap between NMF METcor gene subsets and iCluster METcor subsets. **(F)** Overlap between iCluster METcor gene subsets and iCluster CNVcor gene subsets.

Based on the CNV data related to the CNVcor genes, the methylation site data related to the METcor genes, and the transcriptome data of the CNVcor and METcor genes, multi-omics clustering analysis was performed using iCluster. The iCluster clustering results showed that the optimal clustering results were 2 groups. iCluster clustering heat maps depicted the distributions of CNVs of CNVcor genes (Figure 4A) and of methylation sites of METcor genes (Figure 4B) in two iClusters, respectively. There was significantly difference in overall survival between iC1 and iC2 (*p* < 0.0001; Figure 4C). There were high proportions of overlapping samples between NMF CNVcor and iCluster CNVcor gene clustering subsets (Figure 4D), between NMF METcor and iCluster METcor gene clustering subsets (Figure 4E), between iCluster CNVcor and iCluster METcor gene subsets (Figure 4F).

### Differences in Immune Infiltrations Between Two Multi-Omics Molecular Subtypes for Liver Cancer

**Figure 5:**
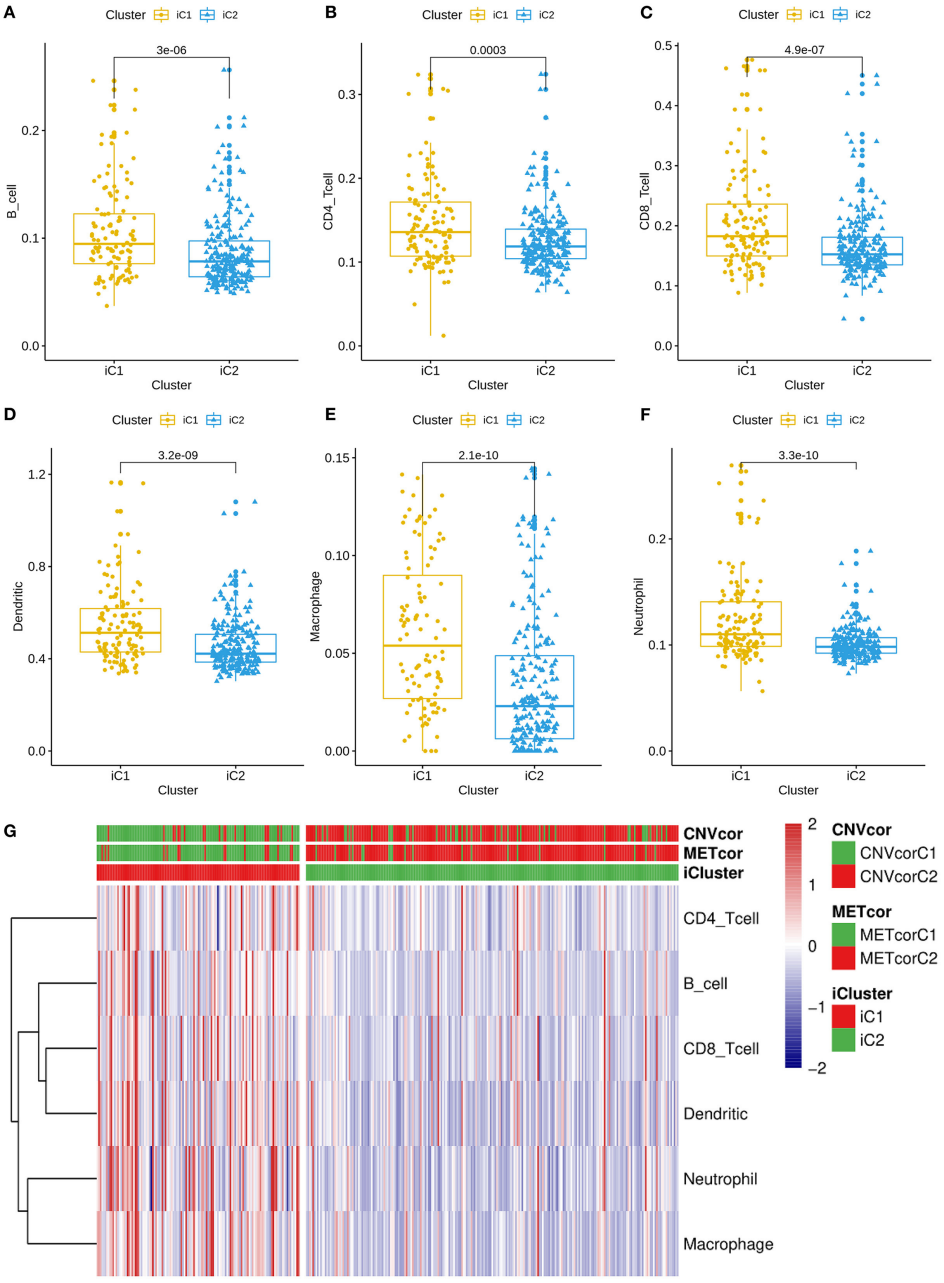
Differences in immune infiltrations between two multi-omics molecular subtypes for liver cancer. Differences in contents of B cells **(A)**, CD4+ T cells **(B)**, CD8+ T cells **(C)**, dendritic cells **(D)**, macrophages **(E)**, and neutrophils **(F)** between iC1 and iC2 subtypes. **(G)** Heatmap for six immune cell scores among all samples.

All genes were clustered into two iClusters. Correlations between genes and immune infiltrations were estimated using TIMER. Intriguingly, we found that the immune scores of iC1 subtype in B cells (*p* = 3e-06; Figure 5A), CD4+ T cells (*p* = 0.0003; Figure 5B), CD8+ T cells (*p* = 4.9e-07; Figure 5C), dendritic cells (*p* = 3.2e-09; Figure 5D), macrophages (*p* = 2.1e-10; Figure 5E) and neutrophils (3.3e-10; Figure 5F) were all significantly higher that of iC2 subtype. Heatmaps depicted that there was significant difference in six immune cell scores between two iClusters (Figure 5G).

### Molecular Features of Gene Subtypes in Liver Cancer

**Figure 6:**
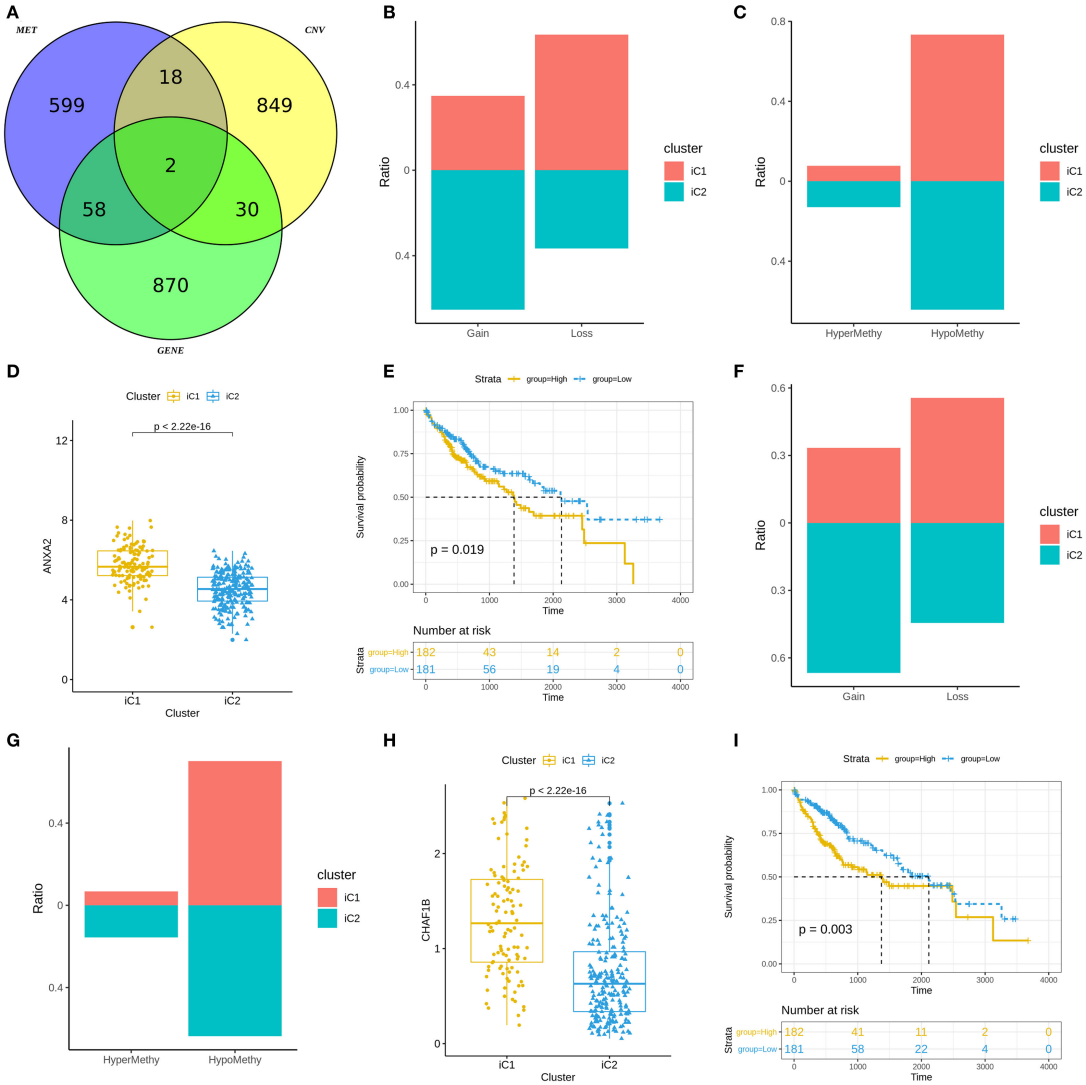
Molecular features of gene subtypes in liver cancer. **(A)** Venn diagram showing differences in CNVs, methylation and mRNA expression between iC1 and iC2 subtypes. **(B)** Proportions of ANXA2 gain and loss in iC1 and iC2 subtypes. **(C)** Proportions of ANXA2 hypermethylation and hypomethylation. **(D)** Box plots showing the differences in ANXA2 expression between iC1 and iC2 subtypes. **(E)** Kaplan-Meier survival curves for ANXA2 expression. **(F)** Proportions of CHAF1B gain and loss in iC1 and iC2 subtypes. **(G)** The proportion of CHAF1B hypermethylation and hypomethylation. **(H)** Box plots showing the differences in CHAF1B expression between iC1 and iC2 subtypes. **(I)** Kaplan-Meier survival analysis results for CHAF1B expression.

We analyzed differences in CNVs (adjusted *p* < 0.01), methylation (adjusted *p* < 0.01) and mRNA expression (|FC|>1.5 and FDR<0.05) between iC1 and iC2 subtypes. Venn diagram showed two genes (including ANXA2 and CHAF1B) differed in CNVs, methylation and mRNA expression between iC1 and iC2 subtypes (Figure 6A). A high proportion of ANXA2 gain in iC2 subtype and its loss in iC1 subtype was found in Figure 6B. Hypomethylated ANXA2 more frequently occurred in iC1 and iC2 subtypes (Figure 6C). Box plots depicted that ANXA2 was significantly up-regulated in iC1 subtype than iC2 subtype (*p* < 2.22e-16; Figure 6D). High ANXA2 expression significantly indicated a poorer prognosis of liver cancer (*p* = 0.019; Figure 6E). There was a higher proportion of CHAF1B gain and a lower proportion of its loss in iC2 compared to iC1 subtype (Figure 6F). CHAF1B hypermethylation more frequently occurred in iC2 subtype (Figure 6G). Higher CHAF1B expression was found in iC2 compared to iC1 subtype (*p* < 2.22e-16; Figure 6H). Its high expression was significantly associated with shorter survival time of patients with liver cancer (*p* = 0.003; Figure 6I).

### Differences in SNVs and Pathways Between Two Multi-Omics Molecular Subtypes for Liver Cancer

**Figure 7:**
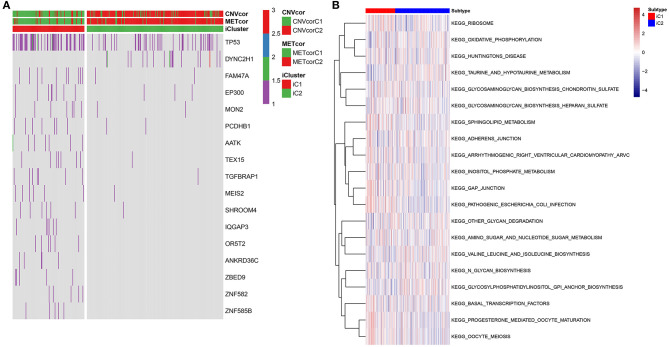
Genetic mutations and enriched pathways in two multi-omics molecular subtypes for liver cancer. **(A)** Differences in genes with single-nucleotide variant (SNV) in iC1 and iC2 multi-omics molecular subtype for liver cancer. Different colors express different numbers of mutations in a gene. **(B)** Differences in enriched signaling pathways between subtypes by GSVA method.

Fisher-exact tests were applied for comparing the differences in SNV locus mutation between two subtypes. Seventeen significant mutated sites with adjusted *p* < 0.01 were screened, as shown in Figure 7A. We found that iC1 subtype had higher frequency mutations than iC2 subtype. We further assessed the correlation between each SNV locus and expression of ANXA2 and CHAF1B. Tables 3, 4 show the top ten SNV loci for ANXA2 and CHAF1B, respectively. Our findings indicated that these SNV loci might affect expression of ANXA2 and CHAF1B. To explore the differences in biological functions between iC1 and iC2 subtypes, GSVA method was applied. As a result, there were distinct differences in metabolism pathways between subtypes such as taurine and hypotaurine metabolism, sphingolipid metabolism, inositol phosphate metabolism, amido sugar and nucleotide sugar metabolism (Figure 7B).

**Table 3:** The top ten most significant associations between ANXA2 expression and SNVs.

**Genes**	**snvGenes**	**SNVs**	**CorrP**	**Corr**
ANXA2	ACAN	p.V2481V	0.00315	0.157184
ANXA2	ADCYAP1R1	p.G46V	0.00315	0.157184
ANXA2	ANAPC1	p.L1872*	0.00315	0.157184
ANXA2	ARFGEF1	p.V494A	0.00315	0.157184
ANXA2	ARHGAP26	p.K475*	0.00315	0.157184
ANXA2	ATP6V0D2	p.M300I	0.00315	0.157184
ANXA2	BRF1	p.Y294C	0.00315	0.157184
ANXA2	C11orf87	p.P168Q	0.00315	0.157184
ANXA2	C2CD4C	p.D129D	0.00315	0.157184
ANXA2	CDH11	p.R50H	0.00315	0.157184

**Table 4:** The top ten most significant associations between CHAF1B expression and SNVs.

**Gene name**	**snvGene**	**SNV**	**Corr**	**CorrP**
CHAF1B	TP53	p.R249S	0.219796	3.30E-05
CHAF1B	ESYT2	p.R569H	0.210288	7.20E-05
CHAF1B	ABCC3	p.T267K	0.158038	0.002988
CHAF1B	ADGRE1	p.C742Y	0.158038	0.002988
CHAF1B	ADGRL4	p.V295A	0.158038	0.002988
CHAF1B	ADH4	p.F182V	0.158038	0.002988
CHAF1B	AKAP10	p.D573Y	0.158038	0.002988
CHAF1B	ALB	p.L609Nfs*33	0.158038	0.002988
CHAF1B	ARHGEF11	p.K1355*	0.158038	0.002988
CHAF1B	ATG2B	p.H211R	0.158038	0.002988

### Validation of ANXA2 and CHAF1B in Liver Cancer Tissues

**Figure 8:**
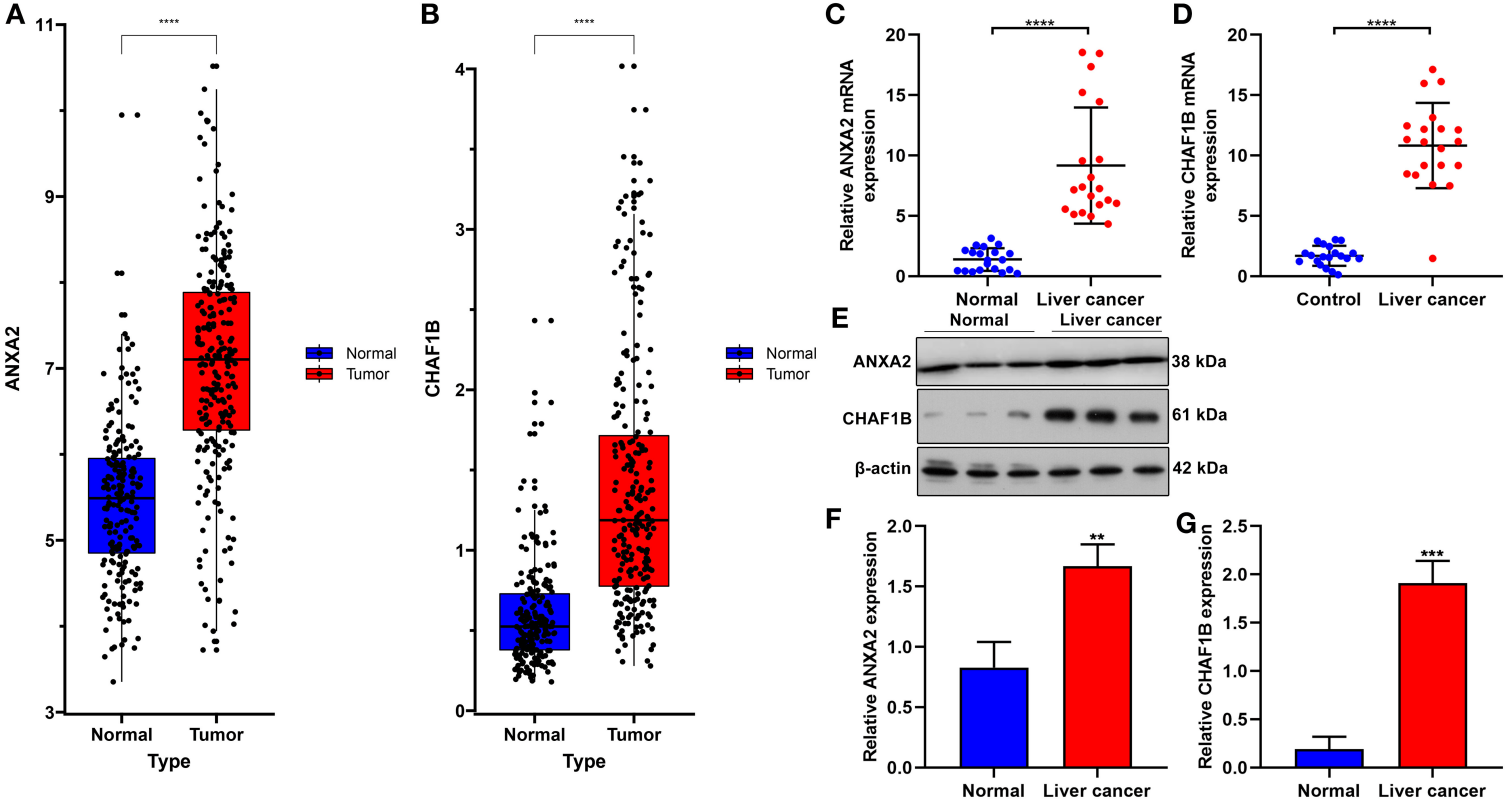
Validation of ANXA2 and CHAF1B mRNAs in liver cancer tissues. **(A,B)** Box plots for ANXA2 and CHAF1B expressions in liver cancer and normal tissues from the ICGC database. **(C,D)** RT-qPCR and **(E–G)** western blot for ANXA2 and CHAF1B expressions in 20 paired liver cancer and normal tissue specimens. ^**^P < 0.01; ^***^P < 0.001; ^****^P < 0.0001.

In the ICGC database, our data confirmed that ANXA2 and CHAF1B were both up-regulated in liver cancer in comparison to normal tissues (Figures 8A,B). Twenty paired liver cancer and normal tissue specimens were harvested in this study. Using RT-qPCR, we validated the mRNA expression of ANXA2 and CHAF1B in liver cancer. The results showed that ANXA2 (Figure 8C) and CHAF1B (Figure 8D) were highly expressed in liver cancer compared to normal specimens, which were consistent with bioinformatics analysis results. Consistently, higher ANXA2 (Figure 8E) and CHAF1B (Figures 8F,G) expressions were found in liver cancer specimens by western blot.

## Discussion

Liver cancer is an aggressive malignant tumor and one of the leading causes of tumor-related deaths (24, 25). Unfortunately, traditional TNM staging can only stratify patients on the basis of clinical manifestations. Despite advances in treatment strategies, effective molecular targets have not been successfully validated. Hence, there is an urgent need to understand the molecular mechanisms and explore therapeutic targets of liver cancer to improve patients' prognosis. With the advances in sequencing technology, it is accessible to obtain large amounts of high-throughput genome sequencing data. Comprehensive analyses about multi-omics data may help conduct accurate management against liver cancer (26–28). Thus, in this study, we integrated multi-omics data from 363 patients with liver cancer to establish two molecular subtypes (iC1 and iC2). Compared with the iC2 subtype, the iC1 subtypes had a worse prognosis. These data emphasize the clinical significance concerning multi-omics analyses of CNVs and methylations in liver cancer. We further characterized the immune cell populations of these two liver cancer subtypes. The scores of the six immune cells of the iC1 subtype were significantly higher than those of the iC2 subtype. In addition, mutation profiles showed that the mutation level of iC1 subtype was markedly higher than that of iC2 subtype, which might lead to poor prognosis of iC1 subtype. Some recent studies have shown that genomics, epigenomics, and transcriptomics play a vital role in tumorigenesis and can predict patients' prognosis (29, 30). Thus, multi-omics studies can help determine tumor heterogeneity, candidate therapeutic targets, and new mechanisms for cancers (22).

By integration of gene expression, CNV gain/loss and hypomethylation/hypermethylation, we identified two prognostic molecular targets, ANXA2 and CHAF1B. Due to the establishment and collection of three data sets and corresponding clinical follow-up information by different organizations, only two overlapping genes in the three data sets may be induced due to internal heterogeneity as well as diversity. These two mRNAs were validated in three independent data sets, suggesting that these genes have universal prognostic significance. Both genes were highly expressed in the iC1 subtype compared to the iC2 subtype. More importantly, their high expression indicated a poorer prognosis. Correlation analysis showed that the mutation site of SNV was significantly correlated with ANXA2 and CHAF1B gene expression. Therefore, assessing the gene expression may be helpful in the diagnoses of early liver cancer. Consistent with previous studies, ANXA2 has been found to be highly expressed in hepatocellular carcinoma (HCC) tissues compared to adjacent normal tissues, furthermore, its high expression is in association with an aggressive phenotype in HCC (31). Highly expressed ANXA2 could induce HCC chemotaxis and metastasis (32), while its knockdown could suppress invasion and migration of liver cancer cells (33). ANXA2 has good diagnostic potential for patients with HBV-related HCC (34). ANXA2 is also involved in the pathogenesis of cardiovascular diseases. For example, both rs11633032 and rs17191344 SNPs can reduce ANXA2 gene expression. Its down-regulation is related to an increased risk of coronary heart disease (35). Also, ANXA2 modulates pulmonary arterial smooth muscle cell proliferation for hepatopulmonary syndrome (36). For CHAF1B, it has been reported that it can promote liver cancer cell migration (37). Thus, in-depth mechanism of these two mRNAs in liver cancer will be probed in further research.

However, several limitations of our study should be pointed out. First, our conclusions were based on retrospective cohorts, and prospective research will be performed to verify these findings. Second, this integrated multi-omics analysis was only based on genomics, epigenomics, and transcriptomics not including proteomics and metabolomics because there were no proteomics and metabolomics data in TCGA database. Third, although our RT-qPCR and western blot results confirmed that ANXA2 and CHAF1B were highly expressed in liver cancer tissues compared to normal specimens, biological functions and mechanisms of ANXA2 and CHAF1B in liver cancer should be further validated.

## Conclusion

In conclusion, we investigated the possible pathogenesis of liver cancer through multi-omics analysis based on genomics, epigenomics, and transcriptomics. Our results suggested that DNA CNV and methylation may play important roles in liver cancer. Furthermore, we identified two clinically relevant molecular subtypes as well as two key biomarkers for liver cancer. These novel mechanisms and clinical classifications may help develop accurate diagnosis and treatments for patients with liver cancer.

## Data Availability Statement

The datasets presented in this study can be found in online repositories. The names of the repository/repositories and accession number(s) can be found in the article/Supplementary Material.

## Ethics Statement

The studies involving human participants were reviewed and approved by the Ethics Committee of The Third Affiliated Hospital of Chongqing Medical University (2019063). The patients/participants provided their written informed consent to participate in this study.

## Author Contributions

DW conceived and designed the study. YS, WX, and QG conducted most of the experiments and data analysis, and wrote the manuscript. QZ, JY, and CL participated in collecting data and helped to draft the manuscript. All authors reviewed and approved the manuscript.

## Conflict of Interest

The authors declare that the research was conducted in the absence of any commercial or financial relationships that could be construed as a potential conflict of interest.

## Supplementary Material

Supplementary Table 1 9161 CNVCor genes for liver cancer.

Supplementary Table 2 6181 METCor genes for liver cancer.

## Abbreviations

CNV: copy number variation
SNV: simple nucleotide variation
TIMER: tumor immune estimation resource
CNVcor: Copy number variation-correlated
METcor: methylation-correlated.

## References

[1] EASL Clinical Practice Guidelines: management of hepatocellular carcinoma. J Hepatol. (2018) 69:182–236. 10.1016/j.jhep.2018.03.019 29628281

[2] Akinyemiju T Abera S Ahmed M Alam N Alemayohu MA Allen C. The burden of primary liver cancer and underlying etiologies from 1990 to 2015 at the global, regional, and national level: results from the global burden of disease study 2015. JAMA Oncol. (2017) 3:1683–91. 10.1001/jamaoncol.2017.3055 28983565 PMC5824275

[3] Siegel RL Miller KD Jemal A. Cancer statistics, 2018. CA Cancer J Clin. (2018) 68:7–30. 10.3322/caac.21442 29313949

[4] Yang JD Roberts LR. Hepatocellular carcinoma: a global view. Nat Rev Gastroenterol Hepatol. (2010) 7:448–58. 10.1038/nrgastro.2010.100 20628345 PMC3926946

[5] Hamdane N Juhling F Crouchet E El Saghire H Thumann C Oudot MA. HCV-induced epigenetic changes associated with liver cancer risk persist after sustained virologic response. Gastroenterology. (2019) 156:2313–29. 10.1053/j.gastro.2019.02.038 30836093 PMC8756817

[6] Perez S Kaspi A Domovitz T Davidovich A Lavi-Itzkovitz A Meirson T. Hepatitis C virus leaves an epigenetic signature post cure of infection by direct-acting antivirals. PLoS Genet. (2019) 15:e1008181. 10.1371/journal.pgen.1008181 31216276 PMC6602261

[7] Zöller B Ji J Sundquist J Sundquist K. Risk of coronary heart disease in patients with cancer: a nationwide follow-up study from Sweden. Eur J Cancer. (2012) 48:121–8. 10.1016/j.ejca.2011.09.015 22023886

[8] Meniconi RL Komatsu S Perdigao F Boelle PY Soubrane O Scatton O. Recurrent hepatocellular carcinoma: a Western strategy that emphasizes the impact of pathologic profile of the first resection. Surgery. (2015) 157:454–62. 10.1016/j.surg.2014.10.011 25633732

[9] Goldman M Craft B Swatloski T Cline M Morozova O Diekhans M. The UCSC cancer genomics browser: update 2015. Nucleic Acids Res. (2015) 43:D812–7. 10.1093/nar/gku1073 25392408 PMC4383911

[10] Kawaguchi K Honda M Yamashita T Okada H Shirasaki T Nishikawa M. Jagged1 DNA copy number variation is associated with poor outcome in liver cancer. Am J Pathol. (2016) 186:2055–67. 10.1016/j.ajpath.2016.04.011 27315779

[11] Hou Y Guo H Cao C Li X Hu B Zhu P. Single-cell triple omics sequencing reveals genetic, epigenetic, and transcriptomic heterogeneity in hepatocellular carcinomas. Cell Res. (2016) 26:304–19. 10.1038/cr.2016.23 26902283 PMC4783472

[12] Lin DC Mayakonda A Dinh HQ Huang P Lin L Liu X. Genomic and epigenomic heterogeneity of hepatocellular carcinoma. Cancer Res. (2017) 77:2255–65. 10.1158/0008-5472.CAN-16-2822 28302680 PMC5413372

[13] Baylin SB Esteller M Rountree MR Bachman KE Schuebel K Herman JG. Aberrant patterns of DNA methylation, chromatin formation and gene expression in cancer. Hum Mol Genet. (2001) 10:687–92. 10.1093/hmg/10.7.687 11257100

[14] Lee ST Wiemels JL. Genome-wide CpG island methylation and intergenic demethylation propensities vary among different tumor sites. Nucleic Acids Res. (2016) 44:1105–17. 10.1093/nar/gkv1038 26464434 PMC4756811

[15] Esteller M. Epigenetic gene silencing in cancer: the DNA hypermethylome. Hum Mol Genet. (2007) 16 Spec No 1:R50–9. 10.1093/hmg/ddm018 17613547

[16] Berdasco M Esteller M. Aberrant epigenetic landscape in cancer: how cellular identity goes awry. Dev Cell. (2010) 19:698–711. 10.1016/j.devcel.2010.10.005 21074720

[17] Hansen KD Timp W Bravo HC Sabunciyan S Langmead B McDonald OG. Increased methylation variation in epigenetic domains across cancer types. Nat Genet. (2011) 43:768–75. 10.1038/ng.865 21706001 PMC3145050

[18] Mermel CH Schumacher SE Hill B Meyerson ML Beroukhim R Getz G. GISTIC2.0 facilitates sensitive and confident localization of the targets of focal somatic copy-number alteration in human cancers. Genome Biol. (2011) 12:R41. 10.1186/gb-2011-12-4-r41 21527027 PMC3218867

[19] Gaujoux R Seoighe C. A flexible R package for nonnegative matrix factorization. BMC Bioinformatics. (2010) 11:367. 10.1186/1471-2105-11-367 20598126 PMC2912887

[20] Li B Severson E Pignon JC Zhao H Li T Novak J. Comprehensive analyses of tumor immunity: implications for cancer immunotherapy. Genome Biol. (2016) 17:174. 10.1186/s13059-016-1028-7 27549193 PMC4993001

[21] Li T Fan J Wang B Traugh N Chen Q Liu JS. TIMER: A web server for comprehensive analysis of tumor-infiltrating immune cells. Cancer Res. (2017) 77:e108–10. 10.1158/0008-5472.CAN-17-0307 29092952 PMC6042652

[22] Hänzelmann S Castelo R Guinney J. GSVA: gene set variation analysis for microarray and RNA-seq data. BMC Bioinformatics. (2013) 14:7. 10.1186/1471-2105-14-7 23323831 PMC3618321

[23] Ritchie ME Phipson B Wu D Hu Y Law CW Shi W. limma powers differential expression analyses for RNA-sequencing and microarray studies. Nucleic Acids Res. (2015) 43:e47. 10.1093/nar/gkv007 25605792 PMC4402510

[24] Chaudhary K Poirion OB Lu L Garmire LX. Deep learning-based multi-omics integration robustly predicts survival in liver cancer. Clin Cancer Res. (2018) 24:1248–59. 10.1158/1078-0432.CCR-17-0853 28982688 PMC6050171

[25] Woo HG Choi JH Yoon S Jee BA Cho EJ Lee JH. Integrative analysis of genomic and epigenomic regulation of the transcriptome in liver cancer. Nat Commun. (2017) 8:839. 10.1038/s41467-017-00991-w 29018224 PMC5635060

[26] Loffler MW Mohr C Bichmann L Freudenmann LK Walzer M Schroeder CM. Multi-omics discovery of exome-derived neoantigens in hepatocellular carcinoma. Genome Med. (2019) 11:28. 10.1186/s13073-019-0636-8 31039795 PMC6492406

[27] Xie Q Fan F Wei W Liu Y Xu Z Zhai L. Multi-omics analyses reveal metabolic alterations regulated by hepatitis B virus core protein in hepatocellular carcinoma cells. Sci Rep. (2017) 7:41089. 10.1038/srep41089 28112229 PMC5253728

[28] Yoo BC Kim KH Woo SM Myung JK. Clinical multi-omics strategies for the effective cancer management. J Proteomics. (2018) 188:97–106. 10.1016/j.jprot.2017.08.010 28821459

[29] Kong L Liu P Zheng M Xue B Liang K Tan X. Multi-omics analysis based on integrated genomics, epigenomics and transcriptomics in pancreatic cancer. Epigenomics. (2020) 12:507–24. 10.2217/epi-2019-0374 32048534

[30] Zheng M Hu Y Gou R Wang J Nie X Li X. Integrated multi-omics analysis of genomics, epigenomics, and transcriptomics in ovarian carcinoma. Aging (Albany NY). (2019) 11:4198–215. 10.18632/aging.102047 31257224 PMC6629004

[31] Wang QS Shi LL Sun F Zhang YF Chen RW Yang SL. High Expression of ANXA2 pseudogene ANXA2P2 promotes an aggressive phenotype in hepatocellular carcinoma. Dis Markers. (2019) 2019:9267046. 10.1155/2019/9267046 30881525 PMC6387700

[32] Li H Wang Y Lu Y Li F. Annexin A2 interacting with ELMO1 regulates HCC chemotaxis and metastasis. Life Sci. (2019) 222:168–74. 10.1016/j.lfs.2019.03.003 30853625

[33] Zhang HJ Yao DF Yao M Huang H Wang L Yan MJ. Annexin A2 silencing inhibits invasion, migration, and tumorigenic potential of hepatoma cells. World J Gastroenterol. (2013) 19:3792–801. 10.3748/wjg.v19.i24.3792 23840117 PMC3699036

[34] Zhang HJ Yao DF Yao M Huang H Wu W Yan MJ. Expression characteristics and diagnostic value of annexin A2 in hepatocellular carcinoma. World J Gastroenterol. (2012) 18:5897–904. 10.3748/wjg.v18.i41.5897 23139605 PMC3491596

[35] Fairoozy RH Cooper J White J Giambartolomei C Folkersen L Wannamethee SG. Identifying low density lipoprotein cholesterol associated variants in the Annexin A2 (ANXA2) gene. Atherosclerosis. (2017) 261:60–8. 10.1016/j.atherosclerosis.2017.04.010 28456096 PMC5446264

[36] Liao L Zheng B Yi B Liu C Chen L Zeng Z. Annexin A2-modulated proliferation of pulmonary arterial smooth muscle cells depends on caveolae and caveolin-1 in hepatopulmonary syndrome. Exp Cell Res. (2017) 359:266–74. 10.1016/j.yexcr.2017.07.020 28729092

[37] Peng X Fu H Yin J Zhao Q. CHAF1B knockdown blocks migration in a hepatocellular carcinoma model. Oncol Rep. (2018) 40:405–13. 10.3892/or.2018.6437 29767268

